# Selinexor reduces the expression of DNA damage repair proteins and sensitizes cancer cells to DNA damaging agents

**DOI:** 10.18632/oncotarget.25637

**Published:** 2018-07-20

**Authors:** Trinayan Kashyap, Christian Argueta, Thaddeus Unger, Boris Klebanov, Sophia Debler, William Senapedis, Marsha L. Crochiere, Margaret S. Lee, Michael Kauffman, Sharon Shacham, Yosef Landesman

**Affiliations:** ^1^ Karyopharm Therapeutics Inc., Newton, MA 02459, USA

**Keywords:** DNA damage repair, selinexor, nuclear export, chemotherapy

## Abstract

**Introduction:**

The goal of this study was to examine the effects of selinexor, an inhibitor of exportin-1 mediated nuclear export, on DNA damage repair and to evaluate the cytotoxic effects of selinexor in combination with DNA damaging agents (DDAs) in cancer cells.

**Results:**

Selinexor reduced the expression of DNA damage repair (DDR) proteins. This did not induce significant DNA damage in tested cell lines. Inhibition of DDR protein expression resulted in enhanced cancer cell death when cells were pretreated with DDAs. In contrast, enhanced cell death was not detected in cells that were pretreated with selinexor then with DDAs. *In vivo*, single-agent selinexor, docetaxel, or cisplatin treatment resulted in 66.7%, 51.5%, and 26.6% tumor growth inhibition (TGI), respectively, in an MDA-MB-231 xenograft model. Consequently, combination treatment with docetaxel or cisplatin followed by selinexor *in vivo* resulted in 93.9% and 103.4% TGI, respectively. Immunohistochemical staining and immunoblot analysis of tumor sections confirmed reduced expression of DDR proteins.

**Conclusion:**

Selinexor treatment inhibited DDR mechanisms in cancer cell lines and therefore potentiated DNA damage-based therapy. The sequential combination of DDAs followed by selinexor increased cancer cell death. This combination is superior to each individual therapy and has a mechanistic rationale as a novel anticancer strategy.

**Methods:**

Cancer cells treated with selinexor ± DDAs were analyzed using reverse phase protein arrays, immunoblots, quantitative PCR and immunofluorescence. Mice bearing MDA-MB-231 tumors were treated with subtherapeutic doses of selinexor, cisplatin, docetaxel and selinexor in combination with either cisplatin or docetaxel. Tumor growth was evaluated for 25 days.

## INTRODUCTION

The export of large macromolecules from the nucleus to the cytoplasm is a highly dynamic and tightly regulated process. Exportin-1 (XPO1, also known as chromosome region maintenance 1 or CRM1) is the best characterized member of the karyopherin family of nuclear transport proteins and is responsible for the export of numerous cargos, including tumor suppressor proteins (TSPs) and regulators of cell growth [1]. Elevated expression of XPO1 mRNA and/or protein has been observed in many types of cancer, and high levels of XPO1 expression are associated with poor prognosis in cancer patients [2–5]. To harness the therapeutic potential of this target, a new class of small molecule inhibitors of XPO1-mediated nuclear export was recently developed, named Selective Inhibitor of Nuclear Export (SINE) compounds.

The lead SINE compound is the orally bioavailable drug selinexor (KPT-330), which is currently under evaluation in phase 2 and 3 clinical trials. Selinexor has demonstrated promising antitumor activity in both solid and hematological cancer types [6–8]. Selinexor and other SINE compounds form slowly reversible covalent bonds with cysteine-528 of the cargo-binding pocket of XPO1. As a result, XPO1 is unable to interact with and export cargo proteins [9], including the TSPs p53, p21, p27, APC, pRb, FOXOs, and the eukaryotic translation initiation factor eIF4E, which facilitates export and translation of proto-oncogenes such as c-Myc, Bcl-2, Bcl-6, cyclins and HSP70. Ultimately, the nuclear enrichment or sequestering of these cargo proteins leads to a reduction of oncogenic translation and the activation of cell cycle arrest, thereby initiating cancer cell death [9].

In healthy cells, cell cycle arrest is needed to control normal tissue growth and to protect the integrity of cellular DNA from mutational assaults, such as free radicals from cellular metabolism, environmental toxins, or cancer treatments, including radiation and chemotherapy [10]. Once DNA damage is detected by a cellular surveillance mechanism, DNA damage response and cell cycle checkpoint proteins induce growth arrest and allow for repair [10]. The repair is executed by nonredundant proteins in a highly coordinated and complimentary manner through five major repair pathways: (1) direct reversal, (2) nucleotide excision repair (NER), (3) base excision repair (BER), (4) mismatch repair (MMR), and (5) recombination repair. However, if the damage cannot be completely repaired, the cell undergoes senescence or apoptosis [11]. Many cancer therapies, including chemo- and radiotherapy, exert their cytotoxic effects by inducing DNA damage [12]. In fact, the extent of DNA damage directly affects the overall cellular response to commonly used cancer therapies [13]. Furthermore, inhibition of different DDR pathways can enhance sensitivity of cancer cells to therapy and increase the level of DNA damage, resulting in synthetic lethality [14]. To this end, several inhibitors of DDR are currently under evaluation in preclinical studies and clinical trials for their ability to enhance DNA damage-induced tumor cell death [14].

In this study, we show that selinexor significantly reduces the expression of DNA damage repair proteins, prevents recovery from DNA damage, and synergizes with DNA damage -inducing chemotherapies to stimulate cell death and reduce tumor size. In addition, the extent of repair protein repression by selinexor correlates with overall sensitivity of cancer cells to the drug. These data suggest that combining selinexor with DNA damage agents (DDA) is a promising strategy for cancer treatment and supports further clinical optimization of dose and schedule to achieve the best therapeutic advantage from the combination treatment of selinexor with DDA.

## RESULTS

### Selinexor reduces the expression of DNA damage repair proteins

**Figure 1:**
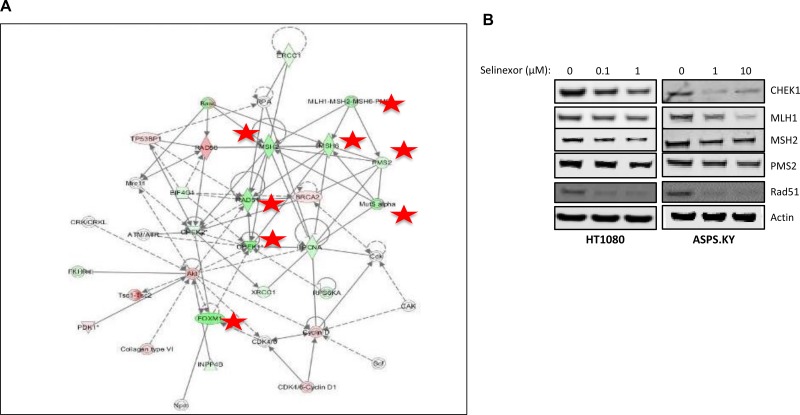
Selinexor reduces the expression of DDR proteins (**A**) Ingenuity pathway analysis (IPA) of 150 proteins from cell lysates of sarcoma cell lines ASPS-KY and HT1080 treated with 10 μM and 1 μM, respectively, of selinexor for 24 hours tested by reverse phase protein array (RPPA) revealed reduction in the expression of 8 proteins (red stars) with a role in DDR. Node shapes represent functional classes of protein products; rectangles with solid lines for cytokines, rectangles with dotted lines for growth factors, triangles for phosphatases, concentric circles for groups or complexes, diamonds for enzymes, and ovals for transcriptional regulators or modulators. (**B**) Western blot of proteins from whole cell lysates of HT1080 cells treated with 0, 0.1, or 1 μM selinexor and ASPS-KY cells treated with 0, 1, or 10 μM selinexor confirmed the RPPA results suggesting down-regulation of CHEK1, MLH1, MSH2, PMS2 and Rad51 protein levels from selinexor treatment in both cell lines.

The selinexor-sensitive fibrosarcoma cell line, HT-1080 (IC_50_ – 100 nM) and the resistant alveolar soft part sarcoma (ASPS) cell line ASPS-KY (IC_50_ > 10 μM) were treated with selinexor at 1 μM for 24 hours or 10 μM for 48 hours. Next, protein expression levels were quantified from whole cell lysates by reverse phase protein array (RPPA) both before and after treatment. RPPA is an antibody-based functional proteomic analysis for both tumor tissue and cultured cells [15]. Ingenuity pathway analysis (IPA) of 150 proteins revealed a reduction in the expression of 8 proteins with roles in DDR: CHEK1, Rad51, MLH1, MSH2, MSH6, PMS2, FOXM1, and Chk2 (Figure 1A; red stars) [16]. The reduction in protein levels was confirmed by western blot analysis using antibodies against the same set of DDR proteins (Figure 1B).

### Selinexor treatment reduces mRNA and protein levels of DDR gene products in solid and hematological cancer cell lines

**Figure 2:**
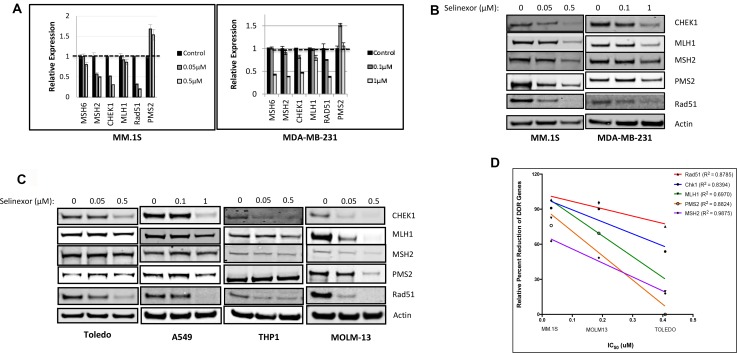
Selinexor suppresses expression of DNA damage gene products at the transcriptional and post-transcriptional levels in both solid and hematological cancer cells (**A**) MM.1S cells were treated with 0 (control), 0.05, or 0.5 μM selinexor and MDA-MB-231 cells were treated with 0, 0.1, or 1 μM selinexor for 24 hours. Real-time PCR indicated that selinexor reduced the transcript levels of MSH6, MSH2, CHEK1, MLH1 and Rad51 in a dose-dependent manner. (**B**) Immunoblots of whole cell lysates from MM.1S cells treated with 0, 0.05, or 0.5 μM selinexor and MDA-MB-231 treated with 0, 0.1, or 1 μM selinexor for 24 hours showed a reduction of the DNA damage repair proteins CHEK1, MLH1, MSH2, PMS2 and Rad51 in a dose-dependent manner. (**C**) Immunoblots of whole cell lysates from A549 cells treated with 0, 0.1, or 1 μM selinexor and Toledo, MOLM-13, and THP-1 cells treated with 0, 0.05, or 0.5 μM selinexor for 24 hours also showed a reduction in the expression of CHEK1, MLH1, MSH2, PMS2 and Rad51 (**D**) The reduction in the levels of DDR proteins is compared for MM.1S, MOLM13 and Toledo. The reduced levels of DDR proteins were found to be directly proportional to the sensitivity of the cells to selinexor.

To confirm that regulation of DDR protein expression by selinexor is not limited to sarcomas, MM.1S (multiple myeloma, selinexor IC_50_ = 30 nM) and MDA-MB-231 (triple negative breast cancer, selinexor IC_50_ = 6.5 μM) cell lines were treated with selinexor for 24 hours, followed by quantitation of mRNA and protein expression by RT-PCR and immunoblotting, respectively. Selinexor reduced the steady-state mRNA levels of key DDR genes in a dose dependent manner: MSH6, MSH2, CHEK1, MLH1 and Rad51, (Figure 2A). Interestingly, while selinexor did not alter the mRNA levels of PMS2 in the two cell lines (Figure 2A), it inhibited PMS2 steady-state protein levels (Figure 2B). These results suggest that selinexor can downregulate the expression of DDR gene products on both the transcriptional and translational level. Selinexor also reduced the protein levels of DDR genes (Figure 2C) in the following cell lines: Toledo (Diffused Large B Cell Lymphoma), selinexor IC_50_ = 410 nM), A549 (non-small cell lung cancer, IC_50_ = 6.5 μM), THP1 (acute monocytic leukemia, IC_50_ = 3 μM) and MOLM13 (acute myeloid leukemia, IC_50_ = 190 nM). We compared the extent of DDR protein reduction with their selinexor sensitivity across these hematological cell lines (Figure 2D). The results suggest that the extent of DDR protein reduction is correlated with overall sensitivity of these cell lines to selinexor (R^2^ between 0.6970 and 0.9875). Analysis of the effects of selinexor on the expression of other DDR genes revealed that treatment also lowered the mRNA steady state levels of other genes, which are responsible for DNA damage surveillance and repair: ATR, CHEK1, ATM, Chk2, BRCA1, BRCA2, RAD51, RAD52, FANCB, PALB2, XRCC1, PARP1, DNA ligase 3, ERCC4, ERCC6 and RPA2 (Supplementary Figure 1).

### Reduction in the levels of DDR genes by selinexor is an early pre-apoptotic event

**Figure 3:**
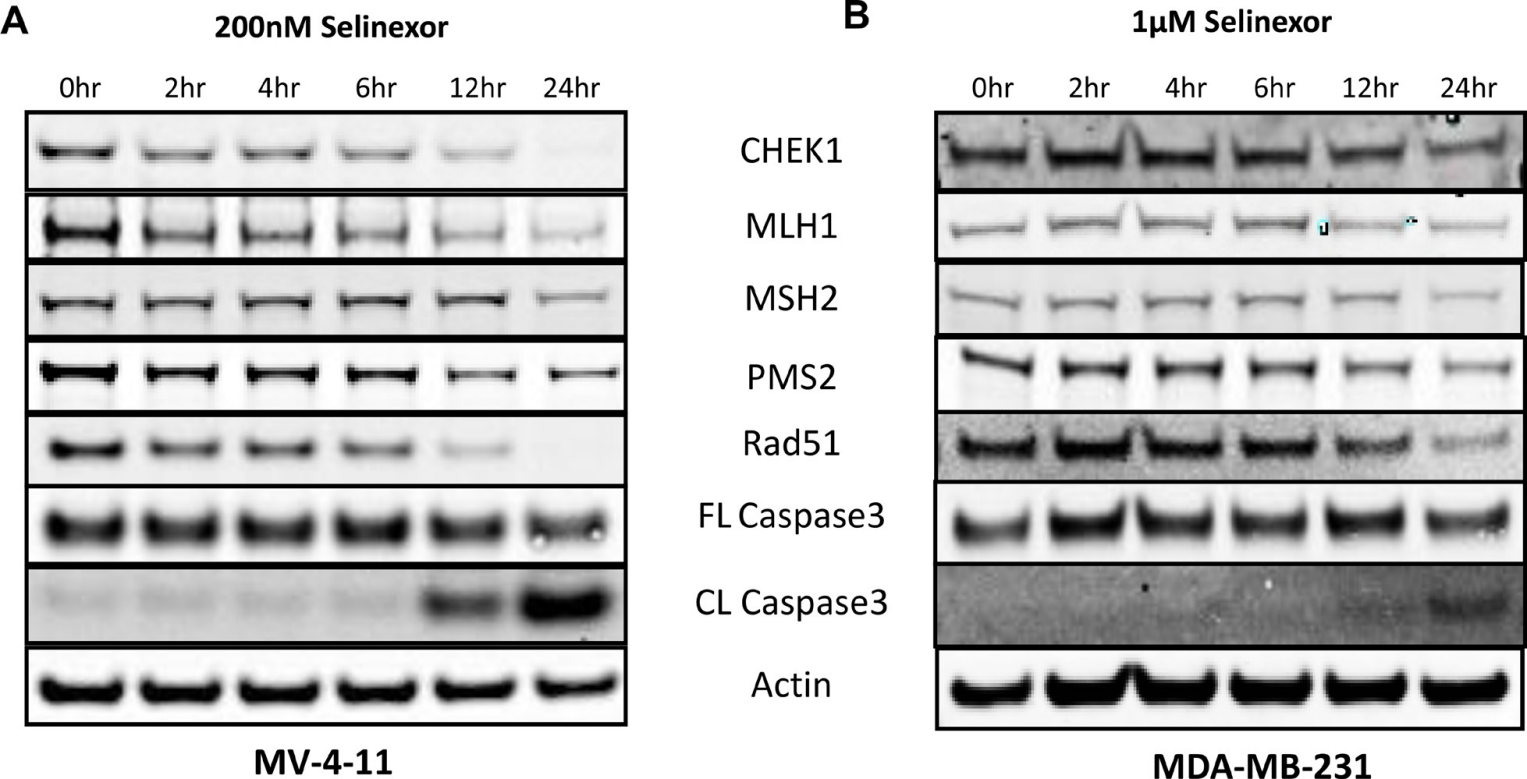
Reduced expression of selinexor dependent expression of DNA damage repair proteins detected prior to cell death Immunoblots of whole cell lysates from (**A**) MV-4-11 and (**B**) MDA-MB-231 cells treated with 200 nM or 1 μM selinexor for 2, 4, 6, 12 and 24 hours. Reduction in the levels of the DDR proteins CHEK1, MLH1, MSH2, PMS2 and Rad51 after selinexor treatment is seen before cell death.

To evaluate when selinexor exposure effects the DDR pathway, MV-4-11 (leukemia, IC_50_ = 120 nM), Figure 3A) and MDA-MB-231 (Figure 3B) cells were treated with 200 nM or 1 μM selinexor, respectively, and sampled at 2, 4, 6, 12, and 24 hours after exposure. Selinexor reduced steady-state protein levels of the DDR genes as early as 2 hours post-exposure while the cytotoxic effects of selinexor, shown by full length (FL) and cleaved (CL) caspase-3, were only evident following prolonged drug exposure of 12 hours or longer. Thus, selinexor interferes with DDR early in the apoptotic pathway.

### Selinexor inhibits recovery after treatment with DNA damaging agents

**Figure 4:**
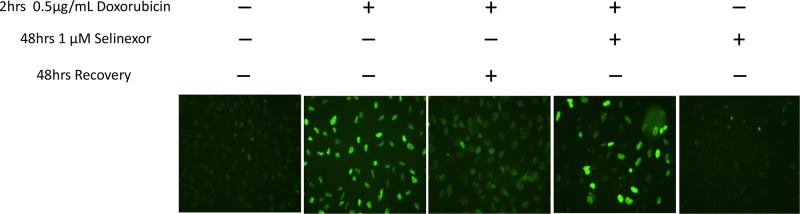
Selinexor blocks recovery from damage caused by DDA Immunofluorescent detection of γH2A.X in U2-0S cells either untreated or treated for 2 hours with 0.5 μg/mL doxorubicin. The cells were then washed and treated with 100 nM selinexor or vehicle for 48 hrs. DNA damage was detected by staining with γH2A.X. DNA damage due to doxorubicin persisted longer in the presence of selinexor. Selinexor alone did not induce DNA damage.

To further elucidate the mechanism of action of selinexor in the DDR pathway, we tested if selinexor induces DNA damage and/or affects DDR. DNA damage by DDA was measured through detection of the DNA-double-strand-break marker γH2A.X immunofluorescence staining after 2 hours of treatment with doxorubicin in osteosarcoma (U-2 OS) cells (Figure 4). When the cells were allowed a 48-hour recovery post-treatment, γH2A.X staining intensity was reduced. However, a 48-hour recovery of cells in media with a suboptimal dose of selinexor, resulted in a higher γH2A.X staining than recovery without selinexor. These data suggest that selinexor interferes with the cell's ability to repair DNA damage. Importantly, treatment with selinexor alone did not induce significant DNA damage under the same conditions (Figure 4).

### Selinexor exhibits synergistic cytotoxic effects in combination with different DNA damaging agents

**Figure 5:**
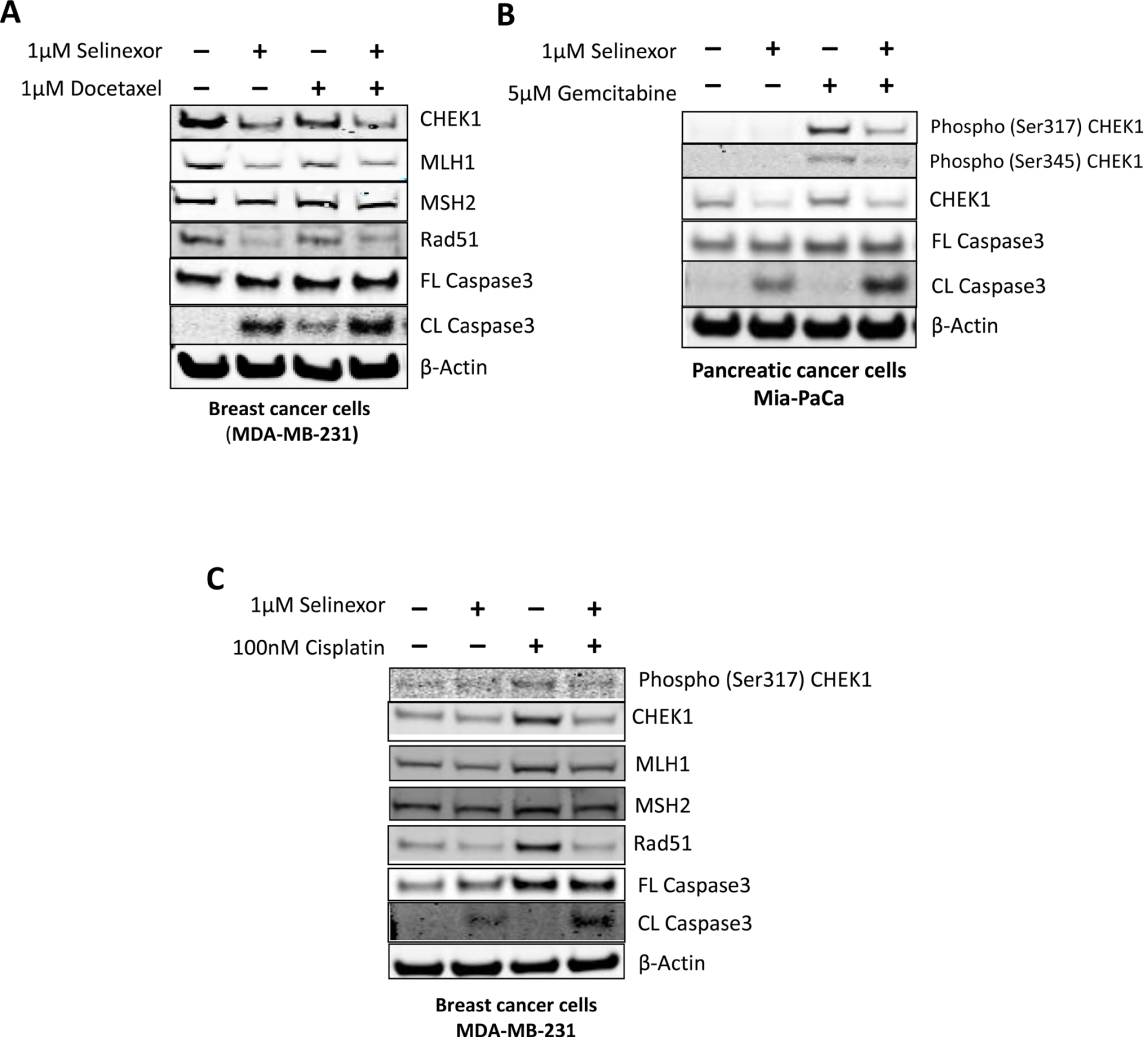
Selinexor exhibits synergistic cytotoxic effects in combination with chemotherapeutic agents Immunoblots of whole cell lysates from MDA-MB-231 cells that were treated with (**A**) 1 μM docetaxel or (**C**) 100 nM cisplatin with or without 1 μM selinexor for 24 hours or (**B**) MiaPaCa-2 cells that were treated with either 5 μM gemcitabine, 1 μM selinexor or the combination for 24 hours. Selinexor inhibited the expression of the DDR proteins CHEK1, MLH1, MSH2 and Rad51 upon exposure to the DDAs and resulted in synergistic cell killing.

Because selinexor reduced DDR protein levels, we examined if the combination of selinexor with different types of DNA damaging agents would result in synergistic cytotoxicity. In order to assess this hypothesis, selinexor was combined with agents that induce single strand breaks (SSB)such as docetaxel (Figure 5A) and gemcitabine (Figure 5B), or with an agent that induces double strand breaks (DSB) such as cisplatin (Figure 5C). When compared to single-agent treatments, all 3 combinations with selinexor showed increased cell death as measured by cleaved caspase-3. In Figure 5A and 5C, reduction of DDR proteins in breast cancer (MDA-MB-231) cells after selinexor treatment is observed in the presence of docetaxel and cisplatin, respectively. Pancreatic cancer (Mia-PaCa2) cells exposed to 5 μM gemcitabine for 24 hours showed increased phosphorylation of CHEK1 at serine 317 and 345, which is indicative of CHEK1 activation [17] (Figure 5B). However, treatment with gemcitabine in the presence of 1 μM selinexor inhibited this activation (phosphorylation) of CHEK1 through reduction of steady-state CHEK1 protein. Thus, selinexor and DDAs together inhibited DDR and enhanced apoptosis as demonstrated by increased caspase- 3 cleavage.

### Selinexor is more cytotoxic to cancer cells when treated after DNA-damaging agents

**Figure 6:**
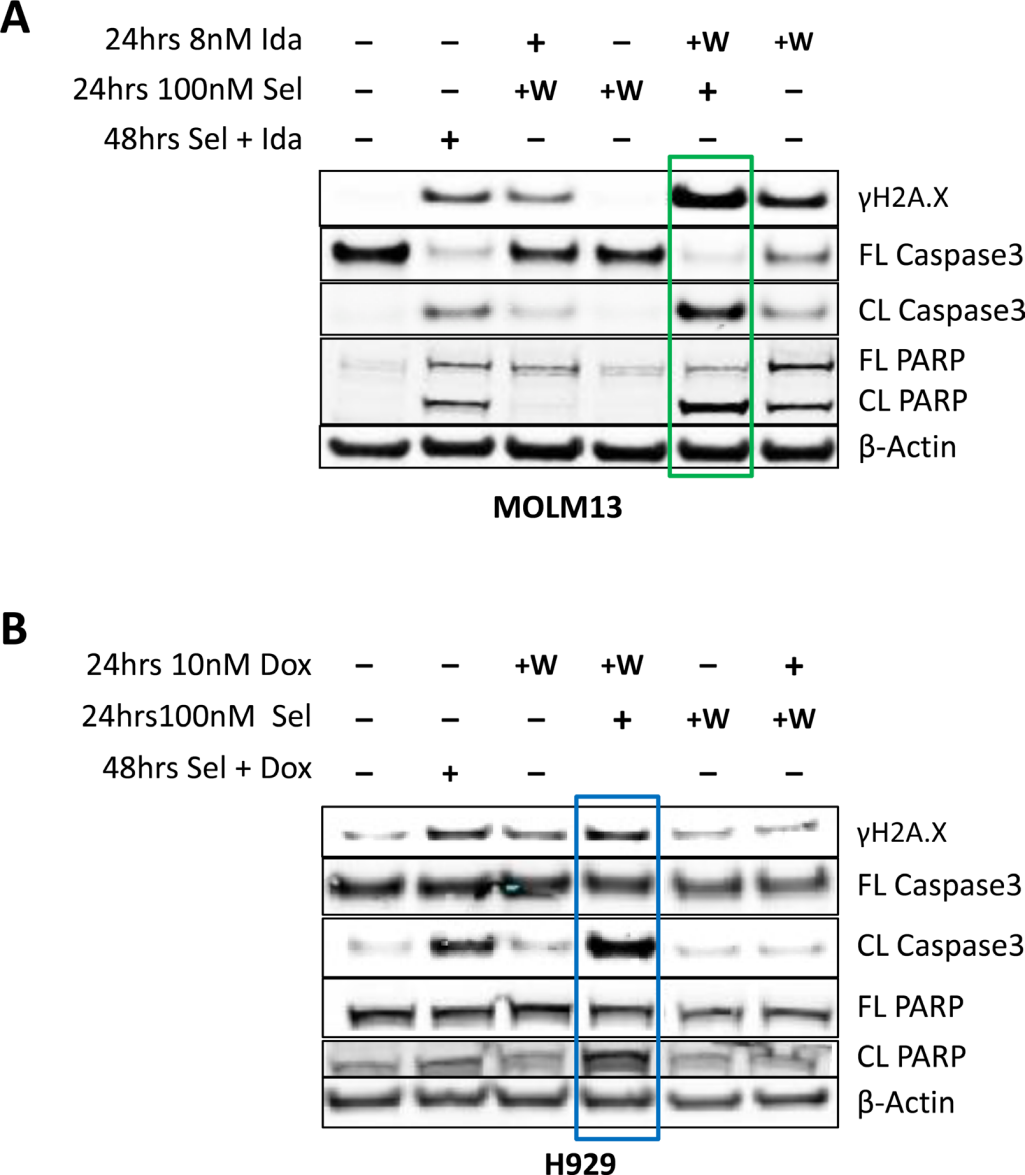
Pre-treatment with DDA followed by selinexor treatment is more cytotoxic than concomitant dosing or pretreatment with selinexor (**A**) Immunoblot analysis of MOLM-13 cells that were either untreated or treated with idarubicin and (**B**) H929 cells that were either untreated or treated with doxorubicin for 24 hours (indicated by “+”), followed by a washout and subsequent treatment with selinexor. The sequence of treatment was then reversed such that cells were first treated with selinexor for 24 hours, washed and then treated with idarubicin or doxorubicin for additional 24 hours. The cells were also treated with each combination together for 48 hours as a control. Pre-treatment of MOLM-13 cells with idarubicin (green box) or H929 (blue box) cells with doxorubicin followed by exposure to selinexor induced more DNA damage and cell death than pre-treatment with selinexor followed by idarubicin or dexorubicin.

In combination therapies, the order of exposure to different drugs can be critical to achieving the best possible outcome. To test if the order of exposure to selinexor and DDAs affected their cytotoxic effect, acute myeloid leukemia cells (MOLM13) were pre-treated with idarubicin for 24 hours followed by 24 hours of selinexor (Figure 6A). The cells showed more DNA damage, as indicated by increased γH2A.X levels and cell death (i.e. increased cleaved PARP-1 and caspase-3 proteins) when compared to 48-hour cotreatment with both compounds or pretreatment with selinexor followed by idarubicin (Figure 6A). A similar result was seen in multiple myeloma (H929) cells treated with doxorubicin then selinexor (Figure 6B). These data support the model where pre-treatment with a DDA followed by exposure to selinexor is more cytotoxic than the reverse sequence or concurrent treatment.

### Combination treatment of selinexor with SSB or DSB DNA-damaging agents shows synergistic anticancer effects in breast cancer xenograft models

**Figure 7:**
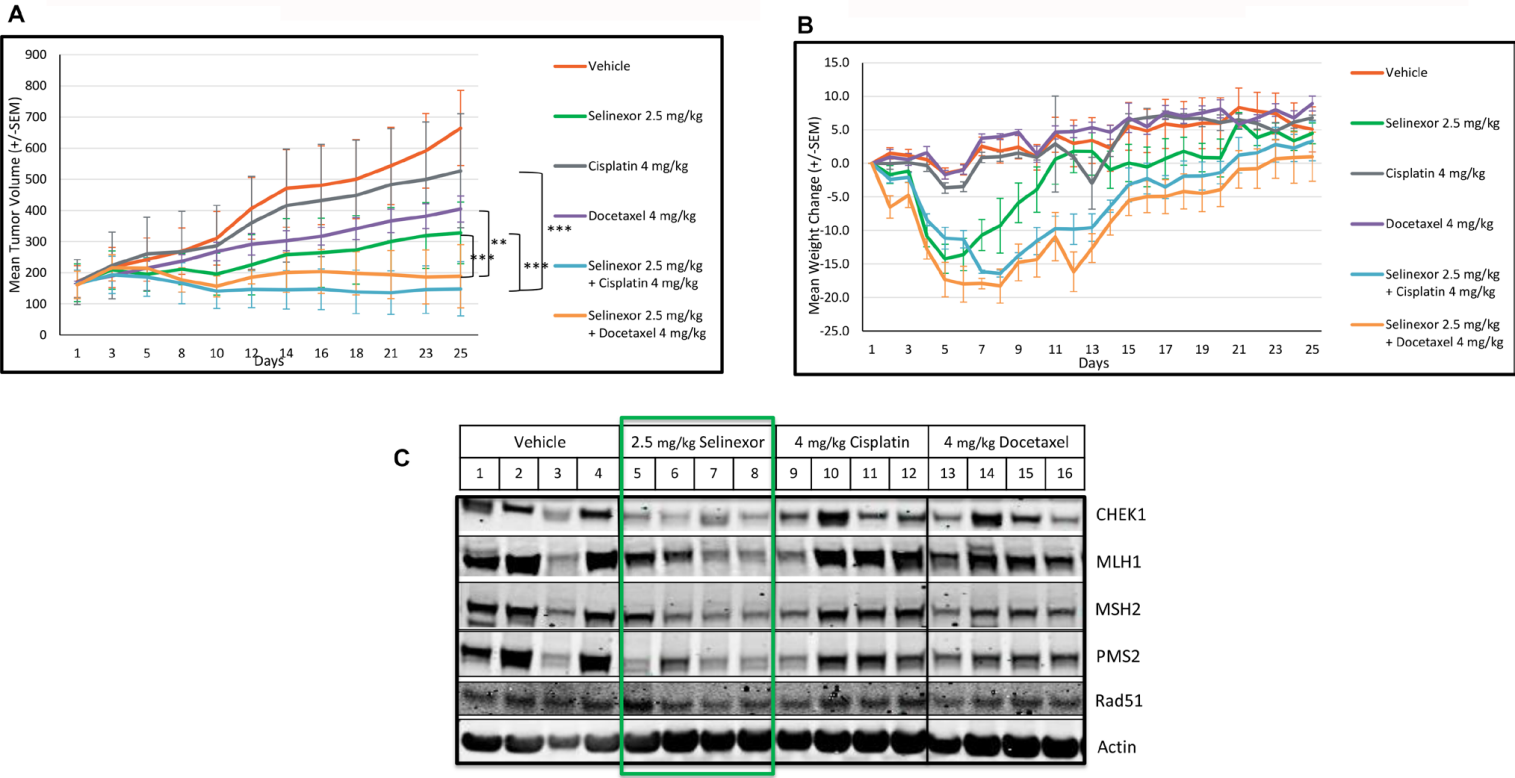
Selinexor demonstrates synergistic anti-tumor effects in combination with cisplatin or docetaxel and inhibits the expression of DDR proteins in an *in vivo* breast cancer model Nu/nu mice were allocated to six groups of 4 mice and treated with vehicle (1), 2.5 mg/kg selinexor (2), 4 mg/kg cisplatin (3), 4 mg/kg docetaxel (4), selinexor in combination with cisplatin (5) or docetaxel (6) for 25 days. For groups V and Vi, selinexor was administered 6 hours after treatment with cisplatin and docetaxel respectively. Selinexor was administered orally, whereas cisplatin and docetaxel were administered by intraperitoneal injection. (**A**) Mean tumor volumes were calculated from the length and width measurements. Group means were calculated and are shown with error bars representing standard error of the mean (SEM) for each group. Combinatory treatments inhibited tumor growth better than each single agent. (**B**) The percent daily weight changes for each animal and the means for each treatment group were calculated. Error bars represent the SEM. There was no significant weight change among the groups at the end of the study. (**C**) At the end of the *in vivo* xenograft study (day 25), excised tumors from the vehicle, selinexor, cisplatin and docetaxel treated groups were assayed either by immunoblots for the expression of DDR proteins. Selinexor, but not cisplatin or docetaxel, reduced the levels of DDR proteins: CHEK1, MLH1, MSH2, PMS2, Rad51.

To further expand on the *in vitro* observations, nu/nu mice engrafted with the breast cancer MDA-MB-231 cells were treated with vehicle, selinexor, cisplatin (DSB agent), docetaxel (SSB agent), or selinexor plus either of the DDAs. The mean tumor volume for the vehicle control group (Group 1) increased from 172 mm^3^ on day 1 to 665 mm^3^ (287%) on day 25. Mice treated with 2.5 mg/kg selinexor, 4 mg/kg cisplatin, and 4 mg/kg docetaxel alone showed a 68% (*p* < 0.05), 28% (not significant) or 53% (*p* < 0.05) tumor growth inhibition (TGI), respectively, when compared to vehicle control. Sequential treatment of 4 mg/kg docetaxel followed by 2.5 mg/kg selinexor or 4 mg/kg cisplatin followed by selinexor resulted in 93.9% (*p* < 0.001) TGI and 103.4% (*p* < 0.001) TGI (9.6% tumor regression), respectively, after 25 days (Figure 7A). Selinexor treatment alone or in combination with DDAs initially resulted in animal weight loss; however, all groups recovered and there were no statistically significant differences in body weight among the treatment groups at the end of the study, as shown in Figure 7B.

### Selinexor inhibits the expression of DDR proteins *in vivo*

To evaluate the impact of selinexor on DDR proteins *in vivo*, the tumors from the vehicle, selinexor, docetaxel, cisplatin, and combination-treated groups were harvested at the end of the study (Figure 7C). Due to the minute size of the tumors from the combination -treated animals, further analysis was not possible. The expression of DDR proteins was examined using immunoblots in tumors from vehicle, selinexor, cisplatin, and docetaxel-treated animals. Selinexor, even at 2.5 mg/kg, lowered DDR protein expression, while cisplatin and docetaxel had no effect on the steady-state levels of the DDR proteins.

## DISCUSSION

Selinexor is a first-in-class, orally bioavailable SINE compound. Selinexor covalently binds to the nuclear export protein XPO1 and inactivates its function. This leads to the nuclear accumulation of key TSPs and the translation initiation factor eIF4E [18]. eIF4E nuclear export is essential for the translation of proto-oncogenes, such as c-Myc, which regulates the transcription of other key DDR genes [19]. DDR is essential for the maintenance of genomic stability and cell survival. Upregulation of several DDR genes, which is observed in several cancers, can increase the efficiency of the DNA repair process and is associated with resistance to chemotherapy and poor prognosis [20]. Consistently, the inhibition of the DDR pathway enhances the therapeutic effects of DNA-damage inducing agents [14].

*In vitro* and *in vivo* studies were used in this study to examine the effects of selinexor in combination with several DDAs: cisplatin, gemcitabine, idarubicin, doxorubicin and docetaxel. DDAs induce different types of damage, which activate specific DDR mechanisms to repair the damage (see Table 1). These agents are frequently used to treat a broad range of cancer indications. The results in this study show that the antitumor effects of DNA damage-inducing chemotherapies are enhanced by selinexor. We have previously described a selinexor-mediated reduction of c-Myc protein levels in several cancers: multiple myeloma [25], mantle cell lymphoma [26], chronic lymphocytic leukemia [27], non-small cell lung cancer [28], esophageal squamous cell carcinoma [29], and acute myeloid leukemia (AML) [30]. In AML, we previously demonstrated that binding of c-Myc to Rad51 and CHEK1 promoters is significantly decreased by selinexor treatment, therefore proposing at least one direct mechanism of action by which selinexor can directly reduce expression of DDR genes [30]. Here we show that selinexor reduced the expression of additional DDR genes and sensitized cancer cells to different chemotherapies *in vitro* and *in vivo*. Importantly, this effect of selinexor on DDR gene product expression is a general phenomenon, not restricted to certain cancer types. Altogether, we show here that selinexor reduced the expression of gene products in five different DNA damage detection and repair mechanisms: (1) DNA damage sensory proteins (ATM, ATR, CHEK1 and Chk2), (2) homologous recombination (Rad51 [31], CHEK1 [17], BRCA1 and BRCA2 [32], Rad52 [33], FANCB [34], PALB2 [35]), (3) mismatch repair (MSH2, MSH6, MLH1, [36]), (4) nucleotide excision repair (ERCC1 [37], ERCC6 [38], RPA2 [39]), and (5) base excision repair (PARP1 [40], XRCC1 and DNA ligase 3 [41]). In addition, selinexor also reduced the expression of the master regulator of DNA damage repair FOXM1, which has roles in the repair mechanism in DNA damage recognition, remodeling, unwinding of chromatin, and excision of damaged DNA [42]. Seven of the gene products (Rad51, CHEK1, MLH1, MSH2, MSH6, PMS2 and PARP1) disrupted by selinexor were the focus of the study and their reduction at the protein level were confirmed. We showed that selinexor inhibited the expression of these genes in a dose-dependent manner in both solid and hematological cancer cell lines. The inhibition is observed at the transcriptional level with the exception of PMS2 which was shown at the translational level only. Importantly, the inhibition of the DDR gene products is independent from cell death, as it is seen as early as 2 hours following selinexor treatment and much earlier than the 12-hour time point when cancer cell death is significant.

**Table 1:** Selinexor inhibits the steady state levels of gene products that regulate DNA damage repar of different mechanisms

	Type of damage	Repair	Indication
Cisplatin	Alkylating agent cross links and induces bulky lesions	Nucleotide excision repair &Homologous recombination	Carcinomas, sarcomas, lymphomas, bladder cancer, cervical cancer and germ cell tumors [21]
Gemcitabine	Antimetabolite incorporates into DNA	Homologous recombination	Pancreatic, breast, ovarian, bladder and non-small cell lung cancer [22]
Doxorubicin & Idarubicin	Anthracyclines inhibit topo II and inhibit transcription, resulting in DSBs	Homologues recombination and non-homologous end joining	Leukemias, lymphomas, breast, stomach, uterine, ovarian, bladder and lung cancer [23]
Docetaxel	Inhibits microtubule dynamics, which disrupts DDR protein trafficking resulting in SSB	Base Excision Repair and Mismatch Repair	Breast, head and, stomach, prostate and non-small cell lung cancer [24]

HT1080 fibrosarcoma cells were treated with 0 (control), 100 or 1000 nM selinexor for 24 hours. Real-Time PCR gene expression assay demonstrates that selinexor inhibits DNA repair protein gene expression of several functional groups: Checkpoint (early response), recombination repair, base excision repair, nucleotide excision repair, and mismatch DNA repair mechanism.

Visualization of the γH2A.X marker was used to detect DNA damage following treatment with DDAs and to examine if selinexor could directly damage the genome. H2A.X, a member of the H2A family of histones, is phosphorylated following a DSB [43] and is frequently used to indicate the presence of genomic insult. The results show that selinexor as a single-agent did not induce the γH2A.X modification and thus did not directly induce significant DNA damage. It was further demonstrated that when cancer cells were allowed to recover from DNA damage, γH2A.X foci return quickly to their basal levels, indicating recovery from genomic insult. However, when cells were exposed to a DDA and then treated with selinexor, γH2A.X foci persisted (up to 48 hours) after the withdrawal of the DSB inducing agent, indicating a delay in the repair process. While γH2A.X modifications occur in response to direct DSBs, they are also the end result of adducts, single strand breaks, replication or transcriptional blocking lesions [43]. This justifies using γH2A.X as a marker while studying cancer chemotherapies that induce different types of DNA damage [43]. These results suggest that the selinexor-related reduction of DDR proteins slowed or blocked the repair process. This is consistent with a previous study that we have performed highlighting the inhibitory effects of selinexor on homologous recombination [30].

Having shown that selinexor could impair or prolong the DDR process, we examined if the selinexor-related delay in repair was observed in the presence of other chemotherapeutic agents and if this delay affected cell viability. The results showed enhanced cytotoxicity and increased apoptosis when selinexor was administered separately and as a follow-up treatment to the DDA. Primary treatment with selinexor followed by treatment with a DDA resulted in lower levels of cytotoxicity. This may have occurred because selinexor inhibits cell cycle progression and induces cell cycle arrest. As a result, cells do not enter into S-phase and initiate DNA replication and are thus less sensitive to chemotherapeutic agents. Therefore, in order to achieve maximal cytotoxicity, DNA damage should be induced prior to the administration of selinexor.

The results and conclusions described here reveal a mechanistic explanation for previous observations that show selinexor enhances the *in vitro* and *in vivo* effects of DNA damage inducing therapies: (1) doxorubicin in multiple myeloma [44], (2) platinum-based chemotherapies in ovarian cancer [45], (3) nucleoside analogues in leukemia and pancreatic cancers [46, 47], and (4) radiation therapy in rectal cancer [48], and (5) unpublished data in non-small cell lung cancer. Radiation therapy, like chemotherapy, can induce many types of DNA damage, including simple lesions, such as base or sugar modifications, DNA crosslinks, single-strand breaks and more complex lesions, such as DNA double-strand breaks [12]. Using rectal cancer models, Ferreiro-Neira *et al*. showed that radiotherapy in combination with selinexor increased apoptosis and decreased the rate of proliferation, when compared to radiotherapy alone, both *in vivo* and *in vitro* [48]. These data suggest that selinexor synergizes with radiation therapy using similar mechanism of DDR inhibition as demonstrated for combination with chemotherapy.

Cancers are heterogeneous diseases which may carry mutations or epigenetic defects that result in elevated activity or deficiencies in DDR pathways. Microsatellite instability, for example, could potentially be used as a marker to identify repair deficiencies and help select the best type of DNA damage repair agent to combine with selinexor when a defect in mismatch repair is identified [36]. Therefore, gaining patient-specific genetic information on DDR genes should aid in the selection of the optimal combination treatment. In the same way, BRCA1/2 deficient tumors have HR DNA repair deficiency; however, in a subset of these tumors, overexpression of the DNA repair protein FANCD2 helps maintain DDR and repair by relying on NHEJ instead of HR [49]. This information could be exploited by inducing DNA damage and inhibiting the DDR with the addition of selinexor. Understanding the genomic background of the tumor will enable treating physicians to customize a combination regimen targeting specific DDR deficiencies. While these preclinical studies strongly suggest an enhanced benefit from the combination treatment of selinexor with DNA damage inducing therapy, future studies should focus on matching a specific DNA damage inducing therapy with selinexor to a specific tumor type harboring mutations in DDR genes with the goal of achieving maximal therapeutic benefit. Moreover, DDAs are not selective for cancer cells and have side effects at therapeutic doses [50]. Adverse effects are also reported in patients treated with selinexor (e.g. weight loss) [6, 8]. The *in vivo* studies showed that combination treatment of low-dose selinexor and low-dose chemotherapeutic agents were synergistic with limited effects on weight loss suggesting that combination treatments may enable efficacy at lower doses and improve tolerability.

In conclusion, the findings presented in this study suggest that the sequential treatment of DNA damage inducing chemotherapeutic agents followed by selinexor results in therapeutic synergy. The combination of these drugs is expected to allow administration of lower doses of each agent, which could mitigate adverse effects of cancer patients in need of effective treatment.

## MATERIALS AND METHODS

### Cell lines and primary samples

MM.1S (#CRL-2974), H929 (#CRL-9068), Toledo (#CRL-2631), THP-1 (TIB-202), A549 (CCL-185), HT1080 (#CCL-121), U-2 OS (#HTB-96), MiaPaCa-2 (#CRL-1420), MDA-MB-231 (HTB-26) were purchased from ATCC (Manassas, VA, USA), MOLM13 cells (#ACC554) were purchased from DSMZ (Braunschweig, Germany) and ASPS-KY (Hoshino *et al*. 2009) were obtained with the permission of Dr. Shunsuke Yanoma. MM.1S, H929, Toledo, THP-1, ASPS-KY and MDA-MB-231 cells were cultured in RPMI-1640 medium (#10-040-CV; Corning). A549 cells were cultured in F12-K medium (#21127-022; Gibco). MiaPaCa-2 cells were cultured in Dulbecco's Modified Eagle's medium (#10-013-CV; Corning). HT1080 cells were cultured in Eagle's Minimum Essential medium (#10-010-CV; Corning). U-2 OS cells were cultured in McCoy's 5A medium (#12330-031; Gibco). Cell media was supplemented with 10% fetal bovine serum, penicillin 10,000 U/mL, and streptomycin 10,000 μg/mL (#15140-122; Gibco).

### Compounds

Selinexor was obtained from Karyopharm Therapeutics. Docetaxel (#S1148), cisplatin (#S1166), idarubicin (#S1228), gemcitabine (#S1714), and doxorubicin (#S1208) were purchased from Selleckchem (Houston, TX, USA).

### Taqman gene assays and antibodies

Real-time PCR Taqman gene probes were purchased from Life Technologies (Carlsbad, CA, USA) (MSH2: Hs00953523_m1; MLH1: Hs00179866_m1; MSH6: Hs00264721_m1; PMS2: HS00241053_m1; Rad51: Hs00153418_m1; CHEK1: Hs00967506_m1). The antibodies for PARP-1 (#9542), Caspase-3 (#9662), Rad51 (#8875), MLH1 (#3515), CHEK1 (#2360), Gamma H2A.X (#9718), MSH2 (#2850) were purchased from Cell Signaling (Danvers, MA, USA); PMS2 (#2251.00.02) and MSH6 (#2203.00.02) antibodies were purchased from Sdix (Newark, DE, USA). Antibodies targeting XPO1 (#sc-5595) and beta-actin (#sc-81178) were purchased from Santa Cruz Biotechnology. The secondary antibodies for western blotting were purchased form LI-COR (Lincoln, NE, USA) and the secondary antibodies for immunofluorescence were purchased from Life Technologies (#A11008).

### Real-time quantitative reverse transcription-PCR

RNA was extracted from cells using the RNeasy Kit (#74106, Qiagen) and reverse transcribed to cDNA using High Capacity cDNA Reverse Transcription Kit (#4368813, Applied Biosystems). mRNA for the indicated genes was quantified using a ViiA7 Real-Time PCR system and analyzed with V1.2 software (Life Technologies).

### Western blotting

Cells were seeded in 6 well plates at a density of 1.5 × 10^6^ (hematological cells) or 0.5 × 10^6^ (solid cells), treated according to the experimental setup, washed with 1X PBS and then lysed with RIPA buffer (#89901, Thermo Scientific) supplemented with protease inhibitors (# 05892791001, Roche) and phosphatase inhibitors (# 04906837001, Roche). Protein levels were quantified using BCA (#23225, Thermo Scientific). 20 μg of each sample was run in 4–12% Bis-Tris Gel (Life Technologies) and later transferred to nitrocellulose membrane using iBlot Gel Transfer Kit (Life Technologies). The membranes were blocked using LI-COR blocking buffer (#927-40000, LI-COR), probed with the indicated antibodies, and analyzed using LI-COR Odyssey.

### DNA damage recovery assay

U-2 OS cells were seeded in 6-well plates at a density of 0.5 × 10^6^ cells/well and allowed to adhere to collagen treated coverslips overnight. The following day, the cells were treated for 2 hours with 0.5 μg/mL doxorubicin. The media was then removed and replaced by media containing DMSO or 1 μM selinexor for 48 hours. The cells were fixed using ice-cold 100% methanol for 15 minutes, washed with 1X PBS, and then blocked/permeabilized using a solution containing 0.1% Tween-20, 0.3 M Glycine, and 1% BSA in 1X PBS. The cells were incubated with the gamma H2A.X (γH2A.X) antibody overnight at 4°C, and then washed 3 times with 1X PBS before incubation with secondary antibody (1:2000) for 1 hour. The cells were washed with 1X PBS, and counterstained with DAPI for 5 minutes and then mounted to a glass slide using Vectashield mounting medium (#H-1400, Vector Laboratories). The coverslips were allowed to dry on the glass slide for a minimum of 4 hours before analysis with a fluorescent microscope.

### Sequential/concomitant combination study

MOLM-13 and H929 cells were first treated with 8 nM idarubicin and 10 nM doxorubicin respectively for 24 hours, followed by treatment with 100 nM selinexor for the next 24 hours. In addition, the order of treatments was reversed (selinexor followed by exposure to idarubicin or doxorubicin). As experimental controls, MOLM13 cells were treated with combination of selinexor and idarubicin and H929 cells were treated with combination of selinexor and doxorubicin, for 48hrs. The cells were harvested and subjected to SDS-PAGE and immunoblot analysis for gamma H2A.X, caspase-3, PARP-1 and beta-actin.

### Immunohistochemistry

Formalin-fixed paraffin-embedded (FFPE) tissue blocks were sectioned at 4 μm, and deparaffinized through three washes in xylene and a decreasing series of ethanol. Antigen retrieval was performed in a steam cooker for 15 minutes in Declere (Cell Marque) working solution. Endogenous hydrogenase was blocked with 3% hydrogen peroxide for five minutes. Slides were incubated in casein-based protein block (Biogenex) for 20 minutes before incubation with the respective antibodies at room temperature for 30 minutes. Slides were then rinsed with buffer and incubated with Amplifier from Hi-Def Polymer Detection Kit (Cell Marque) for 10 minutes at room temperature. Afterwards slides were rinsed with buffer and incubated in DAB chromogen for six minutes at room temperature for color development. The slides were counterstained with Hematoxylin I (Richard Allan Scientific), rinsed in water, and dehydrated through a series of increasing ethanol and three changes of xylene. Slides were mounted on coverslips. Digital images of slides were generated via Aperio AT scanner at 20×. Immunohistochemistry assays were performed on a Biogenex i6000 automated stainer. Masson's Trichrome (Polyscientific) staining was performed manually as per the manufacturer's instructions.

### Xenograft study

Twenty-four nude mice (Taconic Biosciences), aged 7 to 8 weeks were inoculated subcutaneously with 2 × 10^7^ MDA-MB-231 (ATCC # HTB-26) breast adenocarcinoma cells. The mean body weight prior to treatment was 21.9 g (standard deviation ± 1.7 g (CV = 7.7%), range 19.1–24.8 g). Treatment was initiated when the tumors reached a mean volume of 167 mm^3^ (standard deviation ± 89.8 mm^3^, (CV = 54%), range 50–365 mm^3^). Mice were allocated to six groups of four mice such that the mean tumor volume in each group was within the range of 161–172 mm^3^. Mice were treated with vehicle, selinexor, cisplatin, and/or docetaxel as shown in Table 2. For the combination group, 5 and 6, treatment with selinexor followed 6 hours post dosing with the DDAs. Animal weights and condition were recorded daily. Tumors were measured once every two days with micro-calipers, and tumor volume was calculated as (length × width × width)/2. Statistical differences between treatment groups were determined using Mann–Whitney Rank Sum or ANOVA tests with a critical value of *p* < 0.05.

**Table 2:** Xenograft study design

Group	Number of animals	Test article	Dose	Route of administration	Schedule
**1**	4	Vehicle (0.6%Pluronic F-68 and 0.6%PVP K29/32)	0.1 ml/10 g	PO	MWF
**2**	4	Selinexor	2.5 mg/kg	PO	MWF
**3**	4	Cisplatin	4 mg/kg	IP	Q7D
**4**	4	Docetaxel	4 mg/kg	IP	Q7D
**5**	4	CisplatinSelinexor	4 mg/kg2.5 mg/kg	IPPO	Q7DMWF
**6**	4	DocetaxelSelinexor	4 mg/kg2.5 mg/kg	IPPO	Q7DMWF

24 nu/nu mice were allocated to six groups of four mice such that the mean tumor volume in each group was within the range of 161–172 mm^3^. Mice were treated with vehicle, selinexor, cisplatin and docetaxel. Abbreviations: PO, oral; IP, intraperitoneal; MWF, Monday, Wednesday and Friday; Q7D, daily for 7 days.

## SUPPLEMENTARY MATERIALS FIGURE


