# Supplementary material for: Selinexor reduces the expression of DNA damage repair proteins and sensitizes cancer cells to DNA damaging agents
Source: Oncotarget. 2018 Jul 20;9(56):30773–86. doi: 10.18632/oncotarget.25637 (PMC6089403; doi:10.18632/oncotarget.25637)
Supplement: Supplementary file 1 [file oncotarget-09-30773-s001.pdf]

Selinexor reduces the expression of DNA damage repair proteins and sensitizes cancer cells to DNA damaging agents

SUPPLEMENTARY MATERIALS

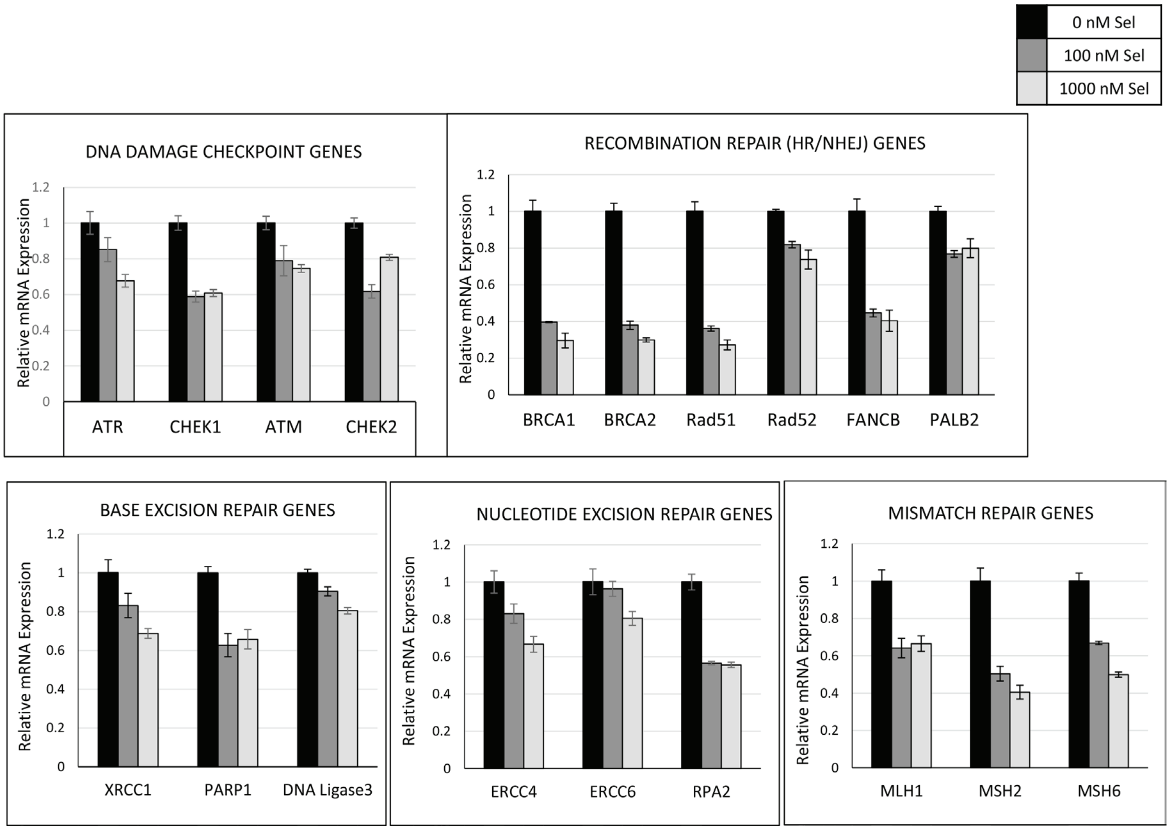

Supplementary Figure 1: Selinexor inhibits the steady state levels of gene products that regulate DNA damage repair of different mechanisms.
